# Engineering prokaryotic gene circuits

**DOI:** 10.1111/j.1574-6976.2008.00139.x

**Published:** 2008-11-05

**Authors:** Konstantinos Michalodimitrakis, Mark Isalan

**Affiliations:** EMBL/CRG Systems Biology Research Unit, Centre for Genomic Regulation (CRG)UPF, Barcelona, Spain

**Keywords:** synthetic biology, gene network, engineering bacteria

## Abstract

Engineering of synthetic gene circuits is a rapidly growing discipline, currently dominated by prokaryotic transcription networks, which can be easily rearranged or rewired to give different output behaviours. In this review, we examine both a rational and a combinatorial design of such networks and discuss progress on using *in vitro* evolution techniques to obtain functional systems. Moving beyond pure transcription networks, more and more networks are being implemented at the level of RNA, taking advantage of mechanisms of translational control and aptamer–small molecule complex formation. Unlike gene expression systems, metabolic components are generally not as interconnectable in any combination, and so engineering of metabolic circuits is a particularly challenging field. Nonetheless, metabolic engineering has immense potential to provide useful biosynthesis tools for biotechnology applications. Finally, although prokaryotes are mostly studied as single cell systems, cell–cell communication networks are now being developed that result in spatial pattern formation in multicellular prokaryote colonies. This represents a crossover with multicellular organisms, showing that prokaryotic systems have the potential to tackle questions traditionally associated with developmental biology. Overall, the current advances in synthetic gene synthesis, ultra-high-throughput DNA sequencing and computation are synergizing to drive synthetic gene network design at an unprecedented pace.

## Introduction

In 2000, four independent studies hallmarked the beginning of the synthetic biology era, by introducing the first synthetic gene circuits. Three of them were purely transcriptional regulation circuits, based on repression and accompanied by rigorous mathematical modelling (Becksei & Serrano, 2000; Elowitz & Leibler, 2000; Gardner *et al.*, 2000), while the fourth engineered an entire enzyme synthesis pathway (Farmer & Liao, 2000). Most of these circuits have already been extensively reviewed (Hasty *et al.*, 2002; Kaern *et al.*, 2003; Sayut *et al.*, 2007), and together they launched the field of gene network engineering. Perhaps for historical reasons, namely the initial string of transcription network papers, the field has been largely dominated by engineering transcription networks. However, it does tend to be easier to couple transcription activation or repression components together, because of their modularity and common mechanism of DNA binding, whereas linking components of different enzyme pathways together is rather more difficult. This may explain why transcription circuits have been studied the most, and thus we will mostly cover examples of these in this review, while also considering a few metabolic systems. There have also been recent advances in combinatorial synthesis and artificial evolution of prokaryotic networks and progress towards engineering spatial patterning in network outputs, rather than simply temporal patterning. With advances in gene synthesis, high-throughput sequencing and computational modelling, the prospects for network engineering have never been so good.

## Transcriptional gene circuits – bottom-up engineering

In prokaryotes, transcription repressors are self-contained units: single proteins with a specific DNA-binding function that leads to transcription downregulation by blocking the binding of the RNA polymerase to a target promoter. By contrast, transcriptional activation is more complicated as there are at least three distinct mechanisms for it in bacteria (Ptashne & Gann, 2002). This is perhaps why the first synthetic gene network studies chose to focus on transcription repressors to build artificial gene circuits, as these single-component effectors were easier to model and understand [exceptions to this include prokaryotic phage polymerases, such as SP6 and T7, which require simple short DNA recognition sites to start transcription, and have thus already been put to good use in synthetic gene networks (Noireaux *et al.*, 2003; Isalan *et al.*, 2005)].

Out of all the possible gene circuits based on repression, the negative feedback loop is the simplest, consisting of a single gene component that downregulates its own production by binding to its promoter (Becksei & Serrano, 2000). Even this example, using the single repressor TetR, is not trivial to understand; whereas the first study on this system focused on the main result that this type of network automatically reduces noise in gene expression, a more recent study has extended the parameter space tested and consequently the picture has become more complex (Dublanche *et al.*, 2006). When there are very low or high amounts of repression in the autoregulatory loop, the noise can actually *increase* because discrete, stochastic expression events can occur, causing output variability. Thus, an important caveat for all synthetic gene network studies is to consider extensive measurement and testing of parameters and variables; unexpected behaviours can arise even in very simple networks.

Repressors were also the building blocks of choice in two other pioneering gene circuits: increasing in network constituents, the genetic toggle switch consists of two repressors mutually repressing each other (Gardner *et al.*, 2000), while the ‘repressilator’ consists of three repressors (destabilized variants of TetR, LacI and CI) controlling each other's expression (Elowitz & Leibler, 2000). The result of the first case is a bistable switch that can flip between two states and the latter is a rather elegant circuit that results in oscillating green fluorescent protein (GFP) output over time. Crucially, the designs for these studies underpinned simple mathematical models. Again, it is important to stress that human intuition is not sufficient to predict the outcomes of even simple networks, even those with a few components, and so computational modelling is essential.

**Fig. 1:**
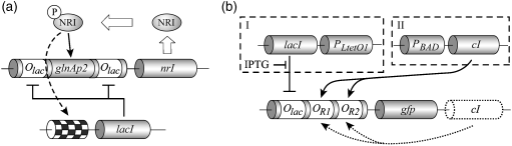
Transcriptional gene circuits. Pointed arrows denote activation, blunt-end arrows repression or inhibition. (a) Atkinson's genetic clock-toggle switch. The checkered promoter upstream of *lacI* can be either the NRI-P responsive *glnK* promoter (dashed arrow), resulting in a genetic clock, or a constitutive promoter, resulting in a toggle switch (Atkinson *et al.*, 2003). (b) A synthetic system for studying different modes of regulation. Box I provides a repression element, Box II provides activation and the dotted *cI* gene provides positive feedback. Using a varying number of these elements, different modes of regulation were studied and used to predict the behaviour of a circuit comprising of all three elements (Guido *et al.*, 2006).

Following these principles of combining computational and ‘wet’ engineering, Atkinson *et al.* (2003) used mathematical models to guide the development of a genetic clock in *Escherichia coli* that exhibited damped oscillations. In contrast to the repressilator, these oscillations were synchronous because, using an isopropyl β-d-1-thiogalactopyranoside (IPTG)-inducible lac repressor, whole bacterial cultures could be reset to different network states. It is worth noting that the system included as part of its architecture the first synthetic prokaryotic-positive feedback loop with a positive regulator: this was based on the Nitrogen Regulator I (NRI) response regulator and an engineered *glnAp2* promoter (Fig. 1a), although a modification of the NRI–NRII two-component signalling pathway was necessary for the loop to be complete.

As in the case of the negative feedback loop by Becskei and Serrano, Guido *et al.* (2006) also used mathematical modelling to study the properties of a synthetic system under different modes of regulation rather than as a guide for the design (Fig. 1b). They used the promoter P_RM_ from λ phage modified to retain only the activating binding sites for CI and an upstream *lac* operator. In this way, they studied the behaviour of the system using GFP as an output, under all types of regulations (no regulation, positive, negative and simultaneously positive and negative) and developed a model to describe it. The success of the model was double, making it one of the best examples of where the interplay of modelling and experiments takes the researcher much further than either approach on its own. On the one hand, it predicted accurately (as verified experimentally) the behaviour of the network when it was expanded to include positive feedback, suggesting that characterization of smaller systems can be useful for predicting the behaviour of larger, more complex networks. On the other hand, it revealed unknown aspects of the network, such as the increase of noise in protein expression levels from high copy number plasmids under arrest of cell growth and division.

While following the output of the circuit is rather straightforward in all the aforementioned cases, sometimes the transcriptional differences in gene expression are too small to be monitored directly, even though they are biologically important. A signal-amplifying circuit can solve this problem and has been applied to reveal previously undetectable responses of Rhl quorum-sensing promoters in *Pseudomonas aeruginosa* (Karig & Weiss, 2005). A common approach is to place a reporter gene directly under the promoter in question. Instead, the ORF for a destabilized CI repressor was placed under the promoter to be tested, which in turn controlled the expression of enhanced yellow fluorescent protein (EYFP) very tightly from a λP_RO12_ promoter. This allowed very low signal variations to be detected, although promoters with different ranges of expression (from basal to fully induced expression) had to be coupled to signal amplifiers with a suitable dynamic operating range. A simplified analogy would be to think of it as a buffer or a pH indicator that can be used only for a range of pH values and not for the entire pH scale. Adjusting the strength of the CI-λP_RO12_, through mutation of the operator sites, resulted in circuits with different dynamic operational ranges. This work neatly exemplifies the crossover between systems biology and synthetic biology, where the quantitative measurements required by the former are enabled by the tinkering approaches of the latter.

Other examples of artificial transcriptional gene circuits are the inverting amplifier based on CI-P_RM_ (Nagaraj & Davies, 2006), the positive feedback loops based on LuxR-P_luxI_ (Sayut *et al.*, 2006) and on CI-P_RM_ (Maeda & Sano, 2006), the transcriptional cascades for studying sensitivity and propagation as a function of network complexity (Hooshangi *et al.*, 2005) and the coupled negative feedback loop (Dublanche *et al.*, 2006). While most of the aforementioned transcriptional gene circuits are single-cell systems, there are also multicellular ones such as the recently published synthetic *E. coli* predator–prey ecosystem based on the combination of two quorum-sensing modules coupled to a suicide gene and its repressor (Balagaddé*et al.*, 2008).

Another common feature of synthetic gene circuits is that the network components are usually designed as monocistronic units (although some circuits use bicistronic operons, the second gene serves as a reporter rather than playing an active role in the network). However, for an increasing number of components, there will be an increasing difficulty to find and encompass several separate transcription units. The use of multicistronic operons is an approach that has already been successfully used by the Keasling group (Martin *et al.*, 2003) for engineering the biosynthetic pathway of the malaria drug precursor amorpha-4,11-diene in *E. coli* where the nine-enzyme pathway was organized into three transcripition units (a tricistronic, a pentacistronic and a monocistronic unit). In fact, this approach led to the development of a new tool for synthetic biology, the tunable intergenic regions (TIGRs, Pfleger *et al.*, 2006). TIGRs are intergenic regions between the genes in an operon containing control elements such as mRNA secondary structures, RNase cleavage sites, ribosome-binding sites (RBS) sequestering sequences, etc., and can vary the relative expression of the coexpressed genes over a 100-fold range, allowing for fine-tuning of the expression level of each gene separately. Although finding the appropriate element to have the desired effect might not be trivial and may require the screening of libraries of such elements, it appears to be a very powerful tool: in this example, it was successfully applied to further fine-tune the tricistronic unit that is involved in the amorphadiene synthesis. This method is likely to be the basis for engineering many other synthetic operons and larger scale networks.

As each year passes, more and more examples of bottom-up engineering are appearing in the literature, indicating that gene network engineering is far more than a passing fad; as the field develops, it will become easier and easier to construct target designs by linking network modules together using a rational design and the Biobrick standard repository of parts (Shetty *et al.*, 2008). Nonetheless, there are alternative ways of engineering gene circuits that require much less knowledge about the components involved: combinatorial engineering and directed evolution.

## Combinatorial synthesis and directed evolution of gene networks

Combinatorial library synthesis has long been one of the cornerstones of protein engineering – which is, in many ways, the forerunner of synthetic biology. In protein engineering, a scaffold such as an antibody chain (Winter *et al.*, 1994) or a zinc finger (Isalan *et al.*, 2001) is randomized in several places, to retain the structure of the scaffold, while gaining new properties such as new binding specificities. By analogy, gene networks can be treated in a very similar way, creating combinations of connections between components, by varying different components at particular positions, while retaining the overall ‘scaffold’ of the network. The difficulty in succeeding with such an approach is that there is rarely a linkage between the genotype and the phenotype that can lead to a selective pressure to select out useful networks; most studies so far have resorted to screening combinatorially synthesized networks one by one.

The combinatorial approach to build a random library of networks was first illustrated by varying connectivity using destabilized TetR, LacI and CI and their respective promoters (Guet *et al.*, 2002). Screening of the library gave some circuits that had the logic functions of NAND, NOR, NOT IF and, most importantly, the study showed that networks with the same topology can have very different behaviours, while networks with similar behaviour can have different topologies. Logic gates have also been studied systematically by the Alon group, in a study where promoter point mutations were mapped to the input functions of the *E. coli* lac operon (Mayo *et al.*, 2006). Even a few mutations can change the input from AND to OR or single input switches, suggesting that regulation is quite plastic and evolvable.

Despite being one of the best-studied systems in all of molecular biology, the lac operon still yields surprises. Dekel & Alon (2005) carried out a study looking at the optimality of the lac network and found that cells can evolve protein levels to optimize growth under certain conditions. On the one hand, this shows that a synthetic gene circuit could be fine-tuned by evolution under appropriate conditions and, on the other, it raises the issue of how stable and robust is the circuit going to be over time in the absence of a selective pressure. This is especially important for engineering whole organisms because some of the engineered properties might be active only under special conditions – they might be ‘de-tuned’ under conditions when there is cost for their production and maintenance but no survival benefit.

Another combinatorial strategy that illustrated how flexible bacterial gene networks can be – and their intrinsic capacity to evolve – came from our own laboratory's work on rewiring bacterial transcription promoters in *E. coli* (Isalan *et al.*, 2008). By systematically linking *c*. 600 promoter regions to transcription or sigma factor ORFs on plasmids, new links were added to the global cellular transcription network. Surprisingly, most added links were well tolerated, showing no growth defects. This was true for even highly connected ‘hub-genes’, which were still amenable to rewiring, indicating that the network may somehow buffer reconnective changes. The panel of altered network constructs was treated as a combinatorial library for selecting out networks with particular properties, such as improved capacity to survive 50 °C heat shock, or improved longevity in the stationary phase. Interestingly, we found clones with improved survival over the wild type under various selection pressures. This is also borne out by the work of Christ & Chin (2007), who carried out a very similar selection approach on a gene library in *E. coli* and found that single genes, as well as gene networks, can confer heat resistance. The network rewiring approach indicates that not only can large-scale bacterial networks tolerate new connections, but that it can be straightforward to select for new properties that confer a survival advantage.

In the same context of rewiring (but not in a combinatorial manner), Haseltine & Arnold (2008) attempted to rewire the quorum-sensing module from *Vibrio fischeri* in an *E. coli* host and showed that with small changes in network connectivity, the response to the input can be a graded, threshold or bistable gene expression.

A hybrid combinatorial-directed evolution strategy was followed by Atsumi & Little (2004) for engineering synthetic λ phage circuits, initially substituting only one of the two λ phage repressors Cro with LacI and later substituting both: Cro with LacI and CI with TetR (Atsumi & Little, 2006). Similar to Gardner and colleagues, they used combinations of *lac* operators and RBS of varying strength, but instead of testing each construct separately, they used genetic selection to isolate engineered circuits that confer regulatory and phenotypic behaviour similar to wild-type λ phage.

**Fig. 2:**
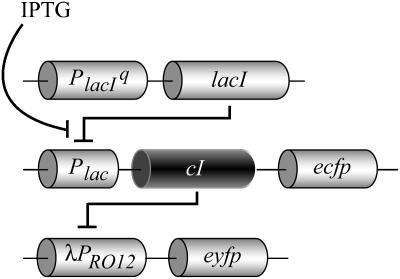
Circuit ‘debugging’ by directed evolution. A nonfunctional circuit is restored using a library of mutant *cI* genes (black cylinder) and screening for functionality (Yokobayashi *et al.*, 2002).

Yokobayashi and colleagues introduced a different approach, a method for ‘debugging’ a nonfunctional synthetic circuit, which can also be used in engineering synthetic circuits. The engineered circuit, depicted in Fig. 2, was meant to couple the output of an IMPLIES gate (defined by the P_lac_/LacI/IPTG interactions and equivalent to ‘NOT LacI OR IPTG’) to an inverter (defined by CI/λP_RO12_). Specifically, LacI was constitutively expressed at high levels from P_lacI_^q^, and so in the absence of IPTG, CI would be repressed and EYFP would be expressed. Addition of IPTG would relieve repression by LacI, leading to expression of CI and repression of EYFP. The circuit, however, was not functional due to a mismatch of the two gates. It was initially ‘debugged’ through a rational design with mutations in the RBS of *cI* and the OR1 operator in λP_RO12_, which were based on simulations of the circuit. However, as this is a rather ‘labour-intensive’ process, as described by the authors, they decided to attempt to restore the function of the circuit through directed evolution: they used error-prone PCR to amplify the *cI* gene, generating a library of mutant circuits in which the only variable is the sequence of the *cI* gene and screened for the desired property. In this way, they isolated mutants that restore circuit function and the mutations actually affect the dimerization of CI more rather than the DNA binding. Thus, directed evolution is a very powerful method with which components of synthetic networks can be fine-tuned to give functional circuits with the desired properties even in more diverse ways than a rational design.

## The ‘RNA connection’

Early synthetic gene circuits were limited mainly to the use of transcription factor-responsive promoter combinations. However, as RNA-based bacterial gene expression regulation is gaining more ground (Brantl, 2004; Grundy & Henkin, 2006), synthetic circuits with RNA components are emerging and are expected to enrich the toolbox of synthetic biologists.

Regulating gene expression *in vivo* with small molecules that bind to RNA was first demonstrated for eukaryotes a decade ago (Werstuck & Green, 1998). They demonstrated that RNA aptamers (RNA molecules that bind small molecules with high affinity and specificity) can bind their target *in vivo*: expressing in *E. coli* an aptamer that binds kanamycin A or tobramycin confers resistance to the respective antibiotic. Based on this, they further showed that incorporation of a dye-binding aptamer into the 5′-untranslated region of a mammalian β-galactosidase expression plasmid allows both *in vitro* and *in vivo* regulation of translation; formation of the dye–aptamer complex represses translation of β-galactosidase mRNA.

However, the use of the RNA aptamer–effector complex for regulation of bacterial gene expression occurred later. Suess *et al.* (2004) designed a synthetic two-domain riboswitch responsive to theophylline and demonstrated its regulatory potential in *Bacillus subtilis*. The riboswitch consisted of a theophylline-binding aptamer and a communication module, that is proposed to perform helix slipping. The riboswitch was placed close to the RBS so that in the absence of theophylline its conformation is such that it interferes with ribosome accessibility. Theophylline binding to the aptamer domain triggers a conformational change in the communication domain, causing a one-nucleotide helix slipping that is sufficient to restore ribosomal accessibility. Almost in parallel, Desai & Gallivan (2004) presented a simple, powerful approach in *E. coli*: they inserted only the theophylline-binding aptamer five nucleotides upstream the RBS of a plasmid-encoded β-galactosidase gene and demonstrated that it is sufficient for efficient and specific positive translational regulation of β-galactosidase. The method can be applied not only for detecting the presence of small molecules inside a cell but also for genetic screens and selections. In fact, such genetic selections can be applied for discovering new synthetic riboswitches that activate translation in response to a compound of interest starting form a library of mutant riboswitches; the latter could be mutants of natural riboswitches or even designed *in silico* (Avihoo *et al.*, 2007).

An interesting alternative genetic selection procedure is the dual selection used by Nomura & Yokobayashi, (2007) who inverted the sign of regulation of a natural thiamine pyrophosphate (TPP) riboswitch from downregulation to activation. Specifically, they generated a plasmid library encoding the *tetA* gene with the TPP aptamer at the 5′ untranslated region at up to 30 random bases upstream the RBS. *TetA* encodes a tetracycline/H^+^ antiporter that confers resistance to tetracycline but also to NiCl_2_. Thus, the same marker gene (*tetA*) was used for selection of both the ON and the OFF states (with tetracycline and NiCl_2_, respectively), minimizing the possibility of false positives. More importantly, the take-home message is that a single aptamer can be used for both positive and negative regulation.

With the RNA aptamer–small molecule strategy, new inputs can be easily implemented in synthetic gene circuits while avoiding some of the obstacles in engineering protein–small molecule interactions. A beautiful network engineering exercise based on this principle is the engineering of the chemotaxis pathway in *E. coli* to respond to a new stimulus, theophylline (Topp & Gallivan, 2007). Although the chemotaxis pathway is a well-studied system, engineering the chemotaxis receptors to recognize new stimuli is not an easy task. In this case, the problem was circumvented by shifting sensing to the RNA level. Specifically, the phosphatase CheZ was cloned in a plasmid with an upstream theophylline-responsive riboswitch and transformed in mutant Δ*cheZ* cells. In the absence of theophylline, the aptamer sequesters the RBS and thus translation is blocked. Because the cells lack CheZ, they are locked into a continuously tumbling state and are thus nonmotile. In the presence of theophylline, the conformation of the aptamer changes, the RBS becomes accessible to the ribosome and CheZ is expressed, not only restoring motility and taxis to wild-type signals but also allowing (pseudo)taxis to theophylline gradients. It is noteworthy that the specificity of the system is remarkable: caffeine, which is structurally very close to theophylline (caffeine can be considered as 7-methyl-theophylline), fails to activate it.

Another approach is the use of RNA aptamers that can cleave part of the RNA transcript (aptazymes). Although such a system is used in nature by *B. subtilis* to actually shut down expression of the enzyme glutamine-fructose-6-phosphate amidotransferase in response to its product, glucosamine-6-phophate (Winkler *et al.*, 2004), a synthetic system has been developed in which cleavage results in activation by removing the part of the transcript that sequesters the RBS (Ogawa & Maeda, 2007).

A different RNA-based strategy was developed where the 5′-untranslated region of the mRNA is designed so that the nascent transcript forms a stem–loop structure sequestering the RBS and thus inhibiting translation (Isaacs *et al.*, 2004). Expression of a highly specific small RNA alters the stem–loop structure, allowing ribosome binding and translation.

In contrast to all the aforementioned methods, which were based on a regulatory RNA component, other RNAs can also be used as a component of synthetic gene circuits. An AND gate was recently constructed in *E. coli* that integrates information from two promoters and controls expression from a third, i.e. expression from the third promoter is possible only when both input promoters are transcriptionally active (Anderson *et al.*, 2007). The output promoter is a T7 promoter and one of the input promoters controls the expression of a T7 RNA polymerase gene that has two internal amber stop codons (TAG) and therefore even if the promoter is active, translation will be prematurely terminated. The second input promoter controls the expression of the amber suppressor tRNA *supD*, which practically converts TAG from a stop to a serine codon. Thus, only when input promoters one AND two are active can the T7 RNA polymerase be produced and can activate the output promoter. The topology of the network allows the use of almost any combination of inducible promoters as input promoters (an obvious limitation would be the basal level/leakiness of the two input promoters) and thus it is one of the most modular synthetic gene circuits yet described.

Logic modules have also been engineered by Rackham & Chin (2005a) who constructed orthogonal ribosome–orthogonal mRNA pairs in *E. coli* so that the engineered ribosomes translate only their cognate mRNAs without any cross-talk with the natural components; neither can the engineered ribosome translate endogenous mRNA nor can the wild-type ribosome translate engineered mRNA. They further constructed logic AND and OR gates in *E. coli* using different combinations of orthogonal ribosome–orthogonal mRNA pairs in the same cell (Rackham & Chin, 2005b; for a detailed discussion, see Isaacs *et al.*, 2006 RNA synthetic biology review).

The potential for RNA in synthetic networks is clearly great because in addition to providing similar levels of control as transcription (i.e. up- or downregulation) it adds the extra dimensions, both spatial and temporal, for separating circuit components from each other. It is likely that cross-talk between such components will result in some very interesting synthetic circuits in the years to come.

## Gene expression–metabolism coupled circuits

As mentioned in the Introduction, the fourth of the pioneering synthetic prokaryotic circuits coupled gene expression to the metabolic state and was used to improve lycopene production in *E. coli* (Farmer & Liao, 2000). Five enzymes were used for the entire lycopene synthesis pathway, with three of the respective genes placed under the *lac* promoter (P_lac_) and only the two genes encoding the rate-controlling enzymes under the regulation of a circuit engineered to be responsive to the metabolic state. The circuit used the response regulator NRI, which, upon activation by phosphorylation, induces expression of the two genes from a minimal *glnAp2* promoter. Coupling the system to metabolism required only deleting the sensor kinase NRII (NtrB), because in its absence NRI becomes responsive (phosphorylated-activated) to acetyl-phosphate (AcP), a small molecule that has been suggested to be an indicator of glucose availability but also able to phosphorylate many response regulators. With this set-up, production was improved threefold not just because there was an increase in the carbon flux to lycopene but because the rechannelling of carbon flux was carried out in a way that did not compromise cell growth (in contrast to overexpressing the two genes, for example).

The Farmer–Liao circuit was also the basis for the development of a synthetic quorum-sensing circuit (Bulter *et al.*, 2004) and a gene expression–metabolic oscillator (Fung *et al.*, 2005), showing that metabolic synthetic biology can be implemented just as well as transcription circuits. In the case of the synthetic quorum-sensing system, acetate was used an input and GFP as an output, which of course required rewiring of acetate and AcP production to reflect cell growth instead of the metabolic state. This was achieved by deleting the phosphate acetyltransferase (*pta*) gene; in the absence of *pta* acetate and AcP, production occurs only through the amino acid biosynthesis pathway and thus becomes proportional to cell growth. Acetate, in the form of acetic acid, diffuses freely across the bacterial membrane into neighbouring cells, where it is phosphorylated to AcP, which in turn phosphorylates NRI and induces GFP expression.

**Fig. 3:**
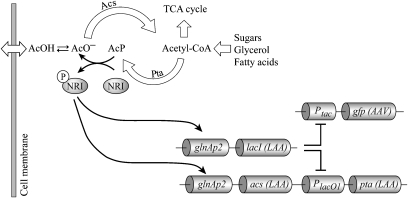
The ‘metabolator’ (Fung *et al.*, 2005) couples metabolism and transcription to generate oscillations. Block arrows denote fluxes, pointed arrows denote activation and blunt-end arrows shows repression. Metabolism leads to accumulation of acetyl-CoA, which, through AcP and NRI, leads to expression of destabilized Acs and repression of destabilized Pta and GFP. As Pta is being degraded, the flux towards AcP decreases until it is insufficient to provide suitable levels of NRI-P for transcription from the glnAp2 promoter. This results in downregulation of Acs and expression of Pta and GFP, closing the oscillatory cycle.

The gene expression–metabolism oscillator, the metabolator (Fung *et al.*, 2005), is a more sophisticated circuit that probably has a more physiological meaning for natural circadian clocks than other synthetic counterparts. It uses genes of destabilized versions of all the proteins involved: plasmid-encoded Pta under P_lac_, chromosomal LacI and plasmid-encoded acetyl-coenzyme A synthetase (ACS) under *glnAp2* and as an output plasmid-encoded GFP under *P*_*tac*_ (Fig. 3). Initially, the levels of acetyl-CoA are higher than those of AcP as it is produced by the metabolism of various compounds and most of it is used by the TCA cycle. Acs and LacI are not expressed, but Pta is. Pta converts the remaining acetyl-CoA to AcP and at a certain point there is enough AcP to phosphorylate NRI and express LacI and Acs. LacI represses Pta expression and because Pta is destabilized flux in that direction will start to decline as Pta is degraded. At the same time, Acs levels will increase as more Acs is being produced. If the glycolytic flux is high and the flux of the reverse reaction (AcP to acetyl-CoA) becomes higher than the sum of fluxes of the forward reaction (acetyl-CoA to AcP) and diffusion of acetic acid out of the cell the pool of acetate-AcP declines, the acetyl-CoA pool increases. This in turn shuts down expression of LacI and Acs, which are degraded, closing the oscillatory cycle. If the glycolytic flux is low, AcP does not accumulate fast enough to change gene expression and the system reaches a steady stable state. This was shown not only by simulations but also experimentally using different carbon sources: cells grown in glucose, fructose or mannose showed oscillatory behaviour while cells grown in glycerol did not. It is noteworthy that addition of acetate in the medium inhibited the oscillations by interfering with the intracellular acetate pool and accumulation of acetate in the medium over time, interfering with the oscillation in long-term experiments.

The engineering of metabolic circuits, while very challenging, has the potential to revolutionize the biotechnology and pharmaceutical industry, because valuable products can be synthesized with an unprecedented degree of control. The challenge over the next few years will be to see whether each project has to be painstakingly developed on an individual basis, or whether a modular framework or a generic ‘toolkit’ can be developed to provide flexible interconnectable enzymatic components.

## Engineering spatial pattern formation

Perhaps one of the most exciting developments in prokaryotic gene circuits in recent years has been the transition from looking at patterns that are achieved in time to ones that make patterns in space. These circuits represent a crossover between single-cell systems, typical of most prokaryotic gene circuits, and multicellular communication systems, such as quorum-sensing and developmental-like patterns.

The simplest form of spatial patterning – crude gradients and banding patterns in transcription–translation mixes – was achieved using magnetic beads to localize gene expression constructs in magnetic chambers (Isalan *et al.*, 2005). Such networks can use either prokaryotic or eukaryotic cell extracts, with T7 or SP6 polymerases for transcription activation, and a variety of transcription repressors to complete the circuits. Whereas this study used positional information predefined in the system (gene expression networks from a spatially localized source), an arguably more sophisticated approach is to use reaction–diffusion-type mechanisms (e.g. Turing patterns; Turing, 1952) to define spots and stripes of gene expression.

**Fig. 4:**
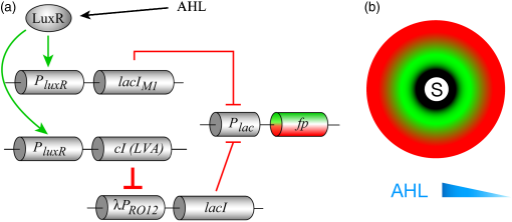
(a) The BD circuit (Basu *et al.*, 2005). The output of the circuit is a FP that is expressed only within a certain range of input (AHL) concentrations. (b) Using a mixture of cells containing a BD circuit with green fluorescent output and cells containing a BD circuit with a red fluorescent output and lower LuxR levels, a bullseye pattern can be formed around a population of ‘sender’ cells (white circle; S) secreting AHL.

The Weiss group has been developing the components for just such an approach, step by step, beginning with a bacterial multicellular system for programmed pattern formation (Basu *et al.*, 2005). The principle behind the system is that ‘sender’ cells produce a signal that induces expression of a reporter fluorescent protein (FP) in ‘receiver’ cells only when the level of the signal is within a specified range of concentrations. This range is determined by a two-component ‘band-detect’ (BD) gene circuit: a low-detect component determines the lowest concentration of the signal that can trigger expression of the output protein and a high-detect component sets the threshold of the signal above which expression of the output protein is shut off. The circuit is shown in Fig. 4. ‘Sender’ cells produce the quorum-sensing signal acetyl-homoserine lactone (AHL) by expressing the *luxI* gene. AHL binding to LuxR in the receiver cells induces expression of the repressors LacI_M1_ (LacI repressor with altered codons to minimize the possibility of recombination) and CI_LVA_ (destabilized CI); the latter represses expression of another allele of LacI. When the AHL concentration is high, induction is strong and LacI_M1_ levels are enough to shut down expression of the FP. When the AHL concentration is too low, neither LacI_M1_ nor CI_LVA_ is expressed but LacI is, again ensuring there is no FP production. At intermediate concentrations of AHL, the levels of LacI_M1_ produced are too low to repress the expression of the FP tightly, but the very strong repressor CI_LVA_ can repress expression of LacI tightly. Thus, FP can be produced only under these conditions. The dynamic operating range, i.e. the range of AHL concentrations that trigger the system, can be set by tuning the LuxR–AHL interaction; using a mixture of cells that contain a BD circuit with a GFP reporter and cells containing a BD circuit with LuxR expressed from a lower copy number plasmid and a dsRed-Express reporter, a bullseye pattern was generated around a group of sender cells, with a red fluorescent outer rim and a green inner rim. The dynamics of pattern formation and its final form depend on the parameters of the circuit, such as the degradation rate of LacI, but also on the spatial arrangement of the sender cells. Even when only one type of BD circuit is used, different patterns can be generated by placing groups of sender cells appropriately (e.g. an ellipse can be formed using two groups of sender cells, a clover using four, etc.).

The above study is a precursor towards fully functional reaction–diffusion-type patterning systems where differential diffusion of system components will ultimately self-organize into patterns of differential gene expression, with no need for defining distinct sender or receiver cells. Therefore, the exciting prospect of seeing an engineered Turing pattern may be closer than ever before, which would further demonstrate the versatility of prokaryotic gene circuits.

## Concluding remarks

Rational design, combinatorial synthesis and directed evolution have all proved to be successful ways of engineering prokaryotic gene networks. The key to a rational design appears to be computational–mathematical modelling: the nonlinearity of molecular interactions is overwhelming for human predictive power and can often result in system behaviours that are quite different from what one might expect. Modelling not only allows predictions of network behaviours but can also determine the parameter regime where a specific type of behaviour is found, thus reducing the number of constructs that need to be made. For example, Rosenfeld *et al.* (2007) recently showed that they could predict accurately the behaviour of a negative feedback loop circuit from the properties of its components. Furthermore, computational tools are emerging that are expected to aid in the design of synthetic circuits (Rodrigo *et al.*, 2007; Sotiropoulos & Kaznessis, 2007; Dasika & Maranas, 2008).

On the other hand, rational design requires considerable high-quality data that are not always available and models with careful attention to details. For example, when attempting to model the cooperativity in the mode of action of a transcription factor, one should not limit the model to cooperative DNA binding but should include the possibility of cooperative stability (i.e. slower degradation rate of the oligomer than the monomer; Buchler *et al.*, 2005). For modelling approaches and pitfalls, we refer the reader to a recent review (Di Ventura *et al.*, 2006).

As far as the quality of the data is concerned, a very interesting point has been raised recently. Nearly all studies use fluorescent reporter genes for quantifying the output of the circuits, but this can be misleading for a number of reasons, including chromophore maturation rate, photobleaching and inclusion body formation. Iafolla *et al.* (2008), using EGFP and P_LtetO1_, showed that the amount of reporter protein that is sequestered in inclusion bodies, and thus not taken into account when measuring fluorescence, can be, under certain conditions, up to *fivefold* more than the soluble measurable fraction, suggesting an additional type of control for the proper interpretation of experimental data.

Even with a state-of-the art model and considerable biochemical data, biological circuits can be context dependent in an unpredictable way. For instance, even using well-characterized components in *Saccharomyces cerevisiae* (*tetO2* operators and *GAL1* promoter), promoters with multiple operators deviated in behaviour from what was expected (Murphy *et al.*, 2007). Moreover, models are usually based on assumptions and simplifications that might not be true for certain systems or conditions. Therefore, previous suggestions (Yokobayashi *et al.*, 2002; Haseltine & Arnold, 2007) of a hybrid of rational design and directed evolution seem to be the most promising and powerful tool. Rational design can guide researchers towards the components and network topology they should use; for example Chen & Wang (2006) developed a method for determining the robust stability of a network under intrinsic fluctuations and identifying the genes that are significantly affected by extrinsic noises, which can be used for robust gene circuit design.

The discovery and analysis of network motifs in natural gene networks (Alon, 2007) can greatly aid in choosing topology of varying complexities. For example, Kim *et al.* (2008) recently studied the effect of coupling two feedback loops and showed that coupled positive feedback loops enhance bistability and signal amplification, coupled negative feedback loops enhance homeostasis and coupled positive–negative feedback loops can attenuate noise.

Nonetheless, the designed system will probably need fine-tuning and this can be greatly facilitated by directed evolution. Directed evolution can be applied in parts of the system and, actually, for complex networks, it is better to characterize and fine-tune a target component using a simpler construct and selection procedure, and then transfer it to the desired network and check for functionality (Alper *et al.*, 2005). Global sensitivity analysis (Feng *et al.*, 2004) could be one way to find candidate components for directed evolution. For designing gene circuits with directed evolution, we refer the reader to Haseltine & Arnold (2007).

The toolbox of synthetic biology is constantly enriched in new types of components, expanding the possibilities for circuit design. Unravelling the principles behind naturally occurring networks has already been successfully applied for recruiting different types of RNAs and there are still more candidates with a great potential for synthetic biology such as robust oscillators based on the cyanobacterial KaiC phosphorylation rhythm (Nakajima *et al.*, 2005; Ito *et al.*, 2007). The latter example is striking because it is an example of a (natural) oscillator system that functions purely at the protein level.

As a final note, it is important to consider the latest advances in technology that are likely to have a direct impact on prokaryotic gene circuit engineering and on synthetic biology as a whole. We already mentioned the use of Biobricks – standard parts that use common restriction enzymes and protocols to link combinations together (Peccoud *et al.*, 2008; Shetty *et al.*, 2008). Alternatively, DNA synthesis technology has always lagged behind DNA sequencing technology, but for the first time it is economically feasible to consider whole gene synthesis (of several kilobasepairs) as an alternative to traditional cloning. A search of the Web quickly reveals several companies that provide this service at a cost of well under a dollar a base. As volumes rise and prices fall further, tinkering with gene network constructs by gene synthesis will become ever more accessible. In parallel, new ultrasequencing technologies can provide over 100 Mbp of sequence for under a thousand dollars. Thus, large combinatorial experiments with many thousands of outputs could, in theory, be characterized by single-shot ultra-high-throughput experiments, as long as ‘genetic bar-coding’ schemes were implemented to allow rapid deconvolution and characterization of network components. Such experiments are very data-rich but fortunately there have been great advances in computational speed, data handling and memory storage that now allow experiments that could not even have been imagined a decade ago. It is hard to predict how our knowledge of gene networks will advance over the next decade, because the scale of the technology revolution makes it hard to comprehend. Doubtless, there are exciting times ahead.

## Statement

Reuse of this article is permitted in accordance with the Creative Commons Deed, Attribution 2.5, which does not permit commercial exploitation.

## References

[b1] Alon U (2007). Network motifs: theory and experimental approaches. Nat Rev Genet.

[b2] Alper H, Fischer C, Nevoigt E, Stephanopoulos G (2005). Tuning genetic control through promoter engineering. P Natl Acad Sci USA.

[b3] Anderson JC, Voigt CA, Arkin AP (2007). Environmental signal integration by a modular AND gate. Mol Syst Biol.

[b4] Atkinson MR, Savageau MA, Myers JT, Ninfa AJ (2003). Development of genetic circuitry exhibiting toggle switch or oscillatory behaviour in *Escherichia coli*. Cell.

[b5] Atsumi S, Little JW (2004). Regulatory circuit design and evolution using phage λ. Genes Dev.

[b6] Atsumi S, Little JW (2006). A synthetic phage λ regulatory circuit. P Natl Acad Sci USA.

[b7] Avihoo A, Gabdank I, Shapira M, Barash D (2007). *In silico* design of small RNA switches. IEEE T Nanobiosci.

[b8] Balagaddé FK, Song H, Ozaki J, Collins CH, Barnet M, Arnold FH, Quake SR, You L (2008). A synthetic *Escherichia coli* predator–prey ecosystem. Mol Syst Biol.

[b9] Basu S, Gerchman Y, Collins CH, Arnold FH, Weiss R (2005). A synthetic multicellular system for programmed pattern formation. Nature.

[b10] Becskei A, Serrano L (2000). Engineering stability in gene networks by autoregulation. Nature.

[b11] Brantl S (2004). Bacterial gene regulation: from transcription attenuation to riboswitches and ribozymes. Trends Microbiol.

[b12] Buchler NE, Gerland U, Hwa T (2005). Nonlinear protein degradation and the function of genetic circuits. P Natl Acad Sci USA.

[b13] Bulter T, Lee SG, Wong WW, Fung E, Connor MR, Liao JC (2004). Design of artificial cell–cell communication using gene and metabolic networks. P Natl Acad Sci USA.

[b14] Chen BS, Wang YC (2006). On the attenuation and amplification of molecular noise in genetic regulatory networks. BMC Bioinformatics.

[b15] Christ D, Chin JW (2007). Engineering *Escherichia coli* heat-resistance by synthetic gene amplification. Protein Eng Des Sel.

[b16] Dasika MS, Maranas CD (2008). OptCircuit: an optimization based method for computational design of genetic circuits. BMC Syst Biol.

[b17] Dekel E, Alon U (2005). Optimality and evolutionary tuning of the expression level of a protein. Nature.

[b18] Desai SK, Gallivan JP (2004). Genetic screens and selections for small molecules based on a synthetic riboswitch that activates protein translation. J Am Chem Soc.

[b19] Di Ventura B, Lemerle C, Michalodimitrakis K, Serrano L (2006). From *in vivo* to *in silico* biology and back. Nature.

[b20] Dublanche Y, Michalodimitrakis K, Kümmerer N, Foglierini M, Serrano L (2006). Noise in transcription negative feedback loops: simulation and experimental analysis. Mol Syst Biol.

[b21] Elowitz MB, Leibler S (2000). A synthetic oscillatory network of transcriptional regulators. Nature.

[b22] Farmer WR, Liao JC (2000). Improving lycopene production in *Escherichia coli* by engineering metabolic control. Nature.

[b23] Feng XJ, Hooshangi S, Chen D, Li G, Weiss R, Rabitz H (2004). Optimizing genetic circuits by global sensitivity analysis. Biophys J.

[b24] Fung E, Wong WW, Suen JK, Bulter T, Lee SG, Liao JC (2005). A synthetic gene-metabolice oscillator. Nature.

[b25] Gardner TS, Cantor CR, Collins JJ (2000). Construction of a genetic toggle switch in *Escherichia coli*. Nature.

[b26] Grundy FJ, Henkin TM (2006). From ribosome to riboswitch: control of gene expression in Bacteria by RNA structural rearrangements. Crit Rev Biochem Mol Biol.

[b27] Guet CC, Elowitz MB, Hsing W, Leibler S (2002). Combinatorial synthesis of genetic networks. Science.

[b28] Guido NJ, Wang X, Adalsteinsson D, McMillen D, Hasty J, Cantor CR, Elston TC, Collins JJ (2006). A bottom-up approach to gene regulation. Nature.

[b29] Haseltine EL, Arnold FH (2007). Synthetic gene circuits: design with directed evolution. Annu Rev Biophys Biomol Struct.

[b30] Haseltine EL, Arnold FH (2008). Implications of rewiring bacterial quorum sensing. Appl Environ Microbiol.

[b31] Hasty J, McMillen D, Collins JJ (2002). Engineered gene circuits. Nature.

[b32] Hooshangi S, Thiberge S, Weiss R (2005). Ultrasensitivity and noise propagation in synthetic transcriptional cascade. P Natl Acad Sci USA.

[b33] Iafolla MA, Mazumder M, Sardana V, Velauthapillai T, Pannu K, McMillen DR (2008). Dark proteins: effect of inclusion body formation on quantification of protein expression. Proteins.

[b34] Isaacs FJ, Dwyer DJ, Ding C, Pervouchine DD, Cantor CR, Collins JJ (2004). Engineered riboregulators enable post-transcriptional control of gene expression. Nat Biotech.

[b35] Isaacs FJ, Dwyer DJ, Collins JJ (2006). RNA synthetic biology. Nat Biotech.

[b36] Isalan M, Klug A, Choo Y (2001). A rapid, generally applicable method to engineer zinc fingers illustrated by targeting the HIV-1 promoter. Nat Biotech.

[b37] Isalan M, Lemerle C, Serrano L (2005). Engineering gene networks to emulate *Drosophila* embryonic pattern formation. PLoS Biol.

[b38] Isalan M, Lemerle C, Michalodimitrakis K, Horn C, Beltrao P, Raineri E, Garriga-Canut M, Serrano L (2008). Evolvability and hierarchy in rewired bacterial gene networks. Nature.

[b39] Ito H, Kageyama H, Mutsuda M, Nakajima M, Oyama T, Kondo T (2007). Autonomous synchronization of the circardian KaiC phosphorylation rhythm. Nat Struct Mol Biol.

[b40] Kaern M, Blake WJ, Collins JJ (2003). The engineering of gene regulatory networks. Annu Rev Biomed Eng.

[b41] Karig D, Weiss R (2005). Signal-amplifying genetic circuit enables *in vivo* observation of weak promoter activation in the Rhl quorum sensing system. Biotechnol Bioeng.

[b42] Kim JR, Yoon Y, Cho KH (2008). Coupled feedback loops form dynamic motifs of cellular networks. Biophys J.

[b43] Maeda YT, Sano M (2006). Regulatory dynamics of synthetic gene networks with positive feedback. J Mol Biol.

[b44] Martin VJJ, Pitera DJ, Withers ST, Newman JD, Keasling JD (2003). Engineering a mevalonate pathway in *Escherichia coli* for production of terpenoids. Nat Biotechnol.

[b45] Mayo AE, Setty Y, Shavit S, Zaslaver A, Alon U (2006). Plasticity of the cis-regulatory input function of a gene. PLoS biol.

[b46] Murphy KF, Balázsi G, Collins JJ (2007). Combinatorial promoter design for engineering noisy gene expression. P Natl Acad Sci USA.

[b47] Nagaraj S, Davies SW (2006). Inverting amplifier genetic circuit performance. Conf Proc IEEE Eng Med Biol Soc.

[b48] Nakajima M, Imai K, Ito H, Nishiwaki T, Murayama Y, Iwasaki H, Oyama T, Kondo T (2005). Reconstitution of circadian oscillatio of cyanobacterial KaiC phosphorylation *in vitro*. Science.

[b49] Noireaux V, Bar-Ziv R, Libchaber A (2003). Principles of cell-free genetic circuit assembly. P Natl Acad Sci USA.

[b50] Nomura Y, Yokobayashi Y (2007). Reengineering a natural riboswitch by dual genetic selection. J Am Chem Soc.

[b51] Ogawa A, Maeda M (2007). Development of a new-type riboswitch using an aptazyme and an anti-RBS sequence. Nucleic Acids Symp Ser (Oxford).

[b52] Peccoud J, Blauvelt MF, Cai Y (2008). Targeted development of registries of biological parts. PLoS ONE.

[b53] Pfleger BF, Pitera DJ, Smolke CD, Keasling JD (2006). Combinatorial engineering of intergenic regions in operons tunes expression of multiple genes. Nat Biotechnol.

[b54] Ptashne M, Gann A (2002). Genes and Signals.

[b55] Rackham O, Chin JW (2005a). A network of orthogonal ribosome–mRNA pairs. Nat Chem Biol.

[b56] Rackham O, Chin JW (2005b). Cellular logic with orthogonal ribosomes. J Am Chem Soc.

[b57] Rodrigo G, Carrera J, Jaramillo A (2007). Genetdes: automatic design of transcriptional networks. Bioinformatics.

[b58] Rosenfeld N, Young JW, Alon U, Swain PS, Elowitz MB (2007). Accurate prediction of gene feedback circuit behavior from component properties. Mol Syst Biol.

[b59] Sayut DJ, Niu Y, Sun L (2006). Construction and engineering of positive feedback loops. Chem Biol.

[b60] Sayut DJ, Kambam PK, Sun L (2007). Engineering and applications of genetic circuits. Mol Biosyst.

[b61] Shetty RP, Endy D, Knight TF (2008). Engineering BioBrick vectors from biobrick parts. J Biol Eng.

[b62] Sotiropoulos V, Kaznessis YN (2007). Synthetic tetracycline-inducible regulatory networks: computer-aided design of dynamic phenotypes. BMC Syst Biol.

[b63] Suess B, Fink B, Berens C, Stenz R, Hillen W (2004). A theophylline responsive riboswitch based on helix slipping controls gene expression *in vivo*. Nucleic Acids Res.

[b64] Topp S, Gallivan JP (2007). Guiding bacteria with small molecules and RNA. J Am Chem Soc.

[b65] Turing AM (1952). The chemical basis of morphogenesis. Philos Trans R Soc Lond B Biol Sci.

[b66] Werstuck G, Green MR (1998). Controlling gene expression in living cells through small molecule–RNA interactions. Science.

[b67] Winkler WC, Nahvi A, Roth A, Collins JA, Breaker RR (2004). Control of gene expression by a natural metabolite-responsive ribozyme. Nature.

[b68] Winter G, Griffiths AD, Hawkins RE, Hoogenboom HR (1994). Making antibodies by phage display technology. Ann Rev Immunol.

[b69] Yokobayashi Y, Weiss R, Arnold FH (2002). Directed evolution of a genetic circuit. P Natl Acad Sci USA.

